# Nuclear Factor κB (NF-κB)–Mediated Inflammation in Multiple Sclerosis

**DOI:** 10.3389/fimmu.2020.00391

**Published:** 2020-03-24

**Authors:** Yifan Zhou, Chunping Cui, Xiaoyu Ma, Wenjing Luo, Song Guo Zheng, Wei Qiu

**Affiliations:** ^1^Department of Neurology, The Third Affiliated Hospital of Sun Yat-sen University, Guangzhou, China; ^2^Department of Internal Medicine, The Ohio State University Wexner Medical Center, Columbus, OH, United States

**Keywords:** NF-κB, multiple sclerosis, demyelination, inflammation, T cells

## Abstract

The nuclear factor κB (NF-κB) signaling cascade has been implicating in a broad range of biological processes, including inflammation, cell proliferation, differentiation, and apoptosis. The past three decades have witnessed a great progress in understanding the impact of aberrant NF-κB regulation on human autoimmune and inflammatory disorders. In this review, we discuss how aberrant NF-κB activation contributes to multiple sclerosis, a typical inflammatory demyelinating disease of the central nervous system, and its involvement in developing potential therapeutic targets.

## Introduction

Nuclear factor κB (NF-κB) comprises a family of transcription factors that coordinate hundreds of genes expression by forming homodimers or heterodimers (http://www.bu.edu/NF-κB/gene-resources/targetgenes/). In mammals, there are five members of NF-κB family: RelA (p65), RelB, c-Rel, p105 (NF-κB1), and p100 (NF-κB2). In most resting cells, NF-κB is sequestered in the cytoplasm through interacting with any of a family of inhibitors of κB (IκB) proteins, such as IκBα, IκBβ, and p100. Upon stimulated signals, IκB kinase (IKK) rapidly phosphorylates IκB and facilitates its ubiquitination and proteasomal degradation, which ultimately enables the entrance of NF-κB into the nucleus and elicits its transcriptional activity (1).

**Figure 1:**
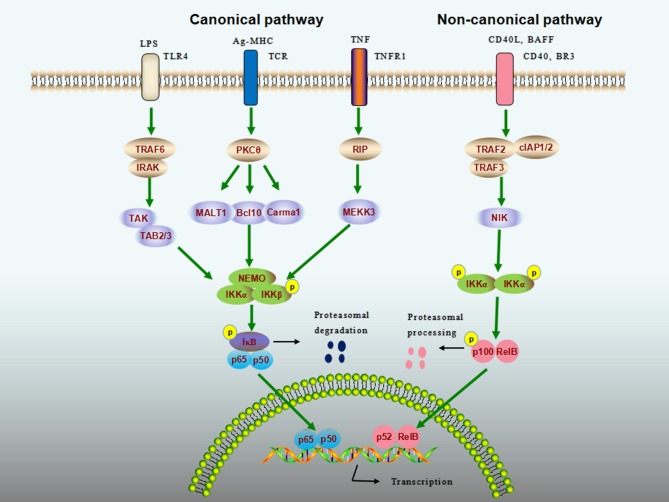
NF-κB signaling cascade. Nuclear factor-κB (NF-κB) activity is stimulated by canonical (classical) and noncanonical (alternative) pathways. The canonical pathway can be activated by extensive numbers of stimuli, such as lipopolysaccharide (LPS), antigens, and tumor-necrosis factor (TNF). The inhibitor of NF-κB (IκB) kinase (IKK) complex that comprises IKKα (IKK1), IKKβ (IKK2), and NF-κB essential modulator (NEMO, also known as IKKγ) is a point of convergence for the canonical pathway, which phosphorylates IκB proteins, allowing the cytoplasmic NF-κB to be released and to enter into the nucleus to elicit transcriptional activity. The noncanonical pathway responds to a different set of ligands, including CD40 ligand (CD40L) and B cell-activation factor (BAFF). Upon binding of these ligands to their cognate receptors, NF-κB-inducing kinase (NIK) specifically phosphorylates IKKα, which processes the p100 into mature p52. The p52 then translocates to the nucleus *via* its dimerization with RelB to activate noncanonical NF-κB target genes.

The general NF-κB signaling cascade can be categorized as canonical (classical) and non-canonical (alternative) pathways (Figure 1). The canonical NK-κB signaling pathway can be induced by extensive numbers of stimuli including Toll-like receptor ligands, proinflammatory cytokines [e.g., tumor necrosis factor α (TNF-α)], and antigens, leading to the activation of IKK complex, which comprises IKKα (IKK1), IKKβ (IKK2), and NF-κB essential modulator (NEMO, also known as IKKγ). The IKK complex then phosphorylates IκB proteins, allowing the cytoplasmic NF-κB dimers (usually p50–p65) to be released from the degraded IκB. The activation of canonical pathway has been shown to mediate a wide range of biological functions within minutes (2, 3). In contrast, the non-canonical NK-κB signaling pathway responds to only a specific set of stimuli such as B cell-activation factor (BAFF), lymphotoxin β, CD40 ligand (CD40L), and receptor activator of nuclear factor kappa-B ligand (4–6). Once stimulated, NF-κB–inducing kinase specifically activates IKKα, which in turn processes the p52 precursor, p100, into p52, and mature p52 subsequently translocates to the nucleus via its dimerization with RelB. Unlike the canonical pathway, this process can be slow and is involved in a limited number of cellular responses (7).

Given that NF-κB affects almost the entire arsenal of immune guardians and immune cells (1), special concern has gradually been focused on the pivotal role of NF-κB dysregulation in many autoimmune inflammatory diseases including multiple sclerosis (MS), systemic lupus erythematosus, and type 1 diabetes.

## MS and Its Animal Models

**Figure 2:**
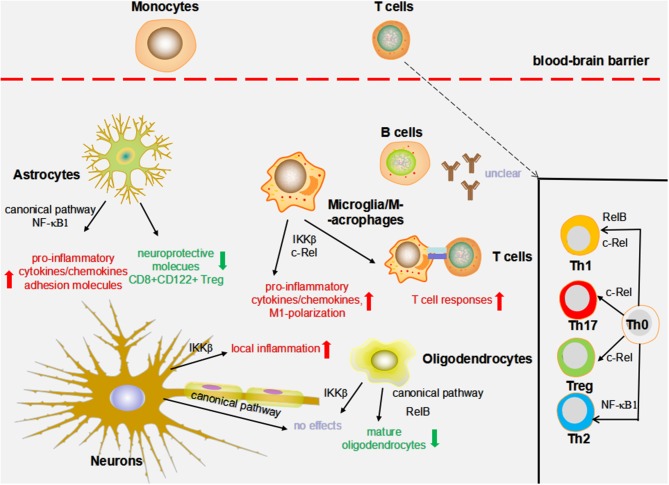
The impact of NF-κB on MS animal models. The effects of nuclear factor-κB (NF-κB) on experimental autoimmune encephalomyelitis (EAE) and cuprizone models are summarized as follows. c-Rel and IKKβ in macrophages/microglia might influence the production of pro-inflammatory cytokines/chemokines, M1 macrophage/microglia phenotype polarization, and T cell immune responses. The deficiency of IKKβ in oligodendrocytes does not alter myelin formation, demyelination, and remyelination; however, blocking RelB and the canonical pathway results in a decreased number of mature oligodendrocytes. NF-κB1 and the canonical pathway are required to augment local inflammation through driving the production of pro-inflammatory mediators and suppressing the levels of neuroprotective molecule adhesion molecules and CD8+ CD122+ regulatory T cells (Tregs). Neuronal IKKβ has been suggested to suppress CNS inflammation. By contrast, conditional deletion of the neuronal NF-κB pathway by the transgenic expression of an IκBα super-repressor did not influence the EAE course. c-Rel is essential in Treg, T helper 1 (Th1), and Th17 differentiation. In addition to c-Rel, Th1 differentiation is also regulated by RelB, whereas NF-κB1 is essential in mediating Th2 responses.

Multiple sclerosis is a multifactorial inflammatory demyelinating disease of the central nervous system (CNS) marked by repeated demyelination and disabling outcomes (5, 8). Although the exact etiology of MS remains unclear, the interaction between predisposing genes and environment triggers MS at a preclinical phase, primarily through affecting the immune system (8). As aberrant peripheral immune cells invade the CNS through disrupted blood–brain barrier (BBB) and induce further inflammation, oligodendrocytes, and neurons are preferentially injured, thereby leading to demyelination and neurodegeneration. Analysis of active human MS lesions demonstrates a complicated immune repertoire that includes but is not limited to lymphocytes, antibodies, cytokines/chemokines, macrophages, microglia, and complement (Figure 2) (9, 10).

Among this intrinsic network, autoreactive T cells against myelin antigens are believed to initiate and augment disease once they migrate into the CNS, where they are reactivated (11). This idea has been reinforced by several lines of indirect evidence: first, myelin-reactive T cells were isolated from both the blood and cerebrospinal fluid of MS patients (12, 13); second, an exacerbated outcome was reported in MS patients treated with a myelin basic protein–derived altered peptide ligand (14); finally, some MS risk variants (e.g., HLA-DRB1^*^1501) were associated with antigen presenting process (15). Despite the fact that myelin-reactive T cells are present in healthy individuals and absent in some MS patients (16, 17), studies have identified several features, such as cytokine secreting profile, activation requirement, and the level of interleukin 2 (IL-2) receptor, which can help distinguish between MS and healthy subjects (18, 19). Further investigations are clearly needed to determine whether self-reactive T cells play a pathogenic role in MS pathogenesis. In this article, we discuss below the prevailing concept that T helper 1 (T_H_1), T_H_17, and CD8^+^ T cells are the major drivers of MS, whereas regulatory T cells (Tregs), perhaps plus T_H_2, confer protective properties.

Most of our understanding on how the immune system regulates MS has originated from experiments performed in experimental autoimmune encephalomyelitis (EAE), a widely used animal model induced by immunizing rodents with myelin proteins/peptides or passively transferring the myelin-reactive T cells to elicit a T cell–mediated autoimmunity (20–22). Experimental autoimmune encephalomyelitis, despite its limitations, mimics many clinical and immunopathological features of human MS. Other models for MS include viral-induced (e.g., Theiler's virus) and toxic-induced (e.g., cuprizone and lysolecithin) demyelinating disorders. Current work exploring the role of NF-κB in MS is based on EAE and the cuprizone model, which is applied to study demyelination and remyelination mechanisms independent of antigen-specific T cells and share some resemblance to those of pattern III MS lesions (23). Demyelination of cuprizone model is characterized by pronounced accumulation of microglia and astrocytes, whereas the contributions of blood-derived immune cells and BBB are minimal (24, 25). Therefore, the role of NF-κB cascade in MS will be discussed, in both humans and animal model (EAE and cuprizone model) with much attention paid to the T cell functioning.

## The Evidence of NF-κB Involvement in Human MS

With the advent of genome-wide association studies and other genetic technologies, a large set of MS susceptibility variants has recently been reported, some of which fall in or near genes that regulate the NF-κB pathway, such as *NFKBIZ* and *RELA* (25–31). Subsequent studies have noted increased levels of NF-κB in total peripheral blood mononuclear cells (PBMCs), CD3^+^/CD4^+^ T cells, and monocytes from patients with MS (32, 33). In addition, CD4^+^ T cells from donors carrying rs228614-G, an MS risk variant proximal to NF-κB1, exhibited increased IκBα degradation and NF-κB p65 nuclear translocation after TNF-α stimulation (34). The altered NF-κB responses were due to the enhanced expression of NF-κB itself, with the rs228614-G variant inducing a 20-fold increase in NF-κB p50 and decrease in several negative regulators of NF-κB (34). Some studies have shown a link between increased NF-κB–related genes in T cells and MS relapse (35, 36). More recently, another MS risk allele, rs7665090-G, was shown to upregulate NF-κB signaling and target genes in astrocytes that increased lymphocytic infiltration and MS lesion size. These findings help to explain how NF-κB may contribute to MS progression in various respects (30). Combined with pathological studies that detected activated NF-κB subunits in macrophages, microglia, oligodendrocytes, astrocytes, and perivascular lymphocytes near or in active MS plaques (37, 38), studies to explore the impact of dysfunctional NF-κB on different cell types on MS would be of interest (Table 1).

**Table 1:** Summary of NF-κB expression in different types of multiple sclerosis.

**References**	**Participants**	**Methods**	**Samples**	**Main conclusions**
Yan et al. (33)	34 RR-MS, 20 SP-MS, 13 PP-MS, 39 HC	WB, Immunocytochemistry, DNA-binding assay	PBMC	Compared to HC, nuclear NF-κB p65 was increased in patients with SP-MS and PP-MS; T cells from all MS subgroups and monocytes from PP-MS showed a higher nuclear p65 proportion than those from HC; the p65 DNA-binding activity in unstimulated PBMC was greater in SP-MS and PP-MS compared to HC.
Eggert et al. (32)	5 RR-MS, 5 PP-MS, 10 SP-MS, 24 HC	DNA-binding assay	PBMC	The level of DNA-binding p50 in 20 MS was significantly higher than in HC but remained unchanged after therapy; the mean level of DNA-binding p65 in 20 MS was proportionate to that of HC, which decreased directly after therapy.
Satoh et al. (35)	6 RR-MS	Microarray analysis	CD3+ T cells	Molecular network analysis suggested a key role of NF-κB in aberrant gene expression in T cells during MS relapse.
Housley et al. (34)	unclear	WB, qPCR, Immunocytochemistry, Luminex	PBMC, plasma	Naïve CD4+ cells from MS patients had higher NF-κB phospho-p65 than those from HC; MS risk variant rs228614 near *NF-κB1* was associated with increased NF-κB signaling; rs1800693 in *TNFR1* was associated with enhanced NF-κB responses to TNF-α and plasma cytokines.
Chen et al. (74)	12 RR-MS, 7 SP-MS, 5 NMO, 9 HC	WB, Flow cytometry	PBMC, CD19+ B cells	B cells from patients with RR-MS and SP-MS exhibited a higher level of NF-κB phospho-p65 after CD40L stimulation compared with HC; after CD40L incubation, no differences in phospho-p65 were found between NMO and HC, but its basal level was much higher in NMO.
Hussman et al. (88)	772 MS, 17376 HC	GWAS	Blood, cell lines, or saliva^*^	A large subset of MS candidate genes was found to interact in a tractable pathway regulating the NF-κB pathway, Th1/Th17 T cell infiltration, and maintenance of regulatory T cells.
Gveric et al. (38)	17 MS, 6 HC	Immunocytochemistry	CNS tissue	In HC white matter, activated NF-κB p65 was found in microglial nuclei, while the c-Rel and p50 subunits and IκBα were restricted to the cytoplasm; in MS active lesions, p65, p50, and IκBα were all present in macrophage nuclei; some hypertrophic astrocytes exhibited nuclear NF-κB p65; perivascular lymphocytes showed nuclear localization of c-Rel.
Bonetti et al. (37)	11 MS, 3 HC	Immunocytochemistry	CNS tissue	In HC white matter and silent MS lesions, the active form of the NF-κB p65 was negligible; up-regulated nuclear NF-κB p65 was observed in active MS lesions on a large proportion of oligodendrocytes and microglia/macrophages.

*MS, multiple sclerosis; RR-MS, relapsing-remitting MS; SP-MS, secondary progressive MS; PP-MS, primary progressive MS; NMO, neuromyelitis optica; HC, healthy controls; WB, Western Blot; PBMC, peripheral blood mononuclear cells; CNS, central nervous system*.

* *DNA from most samples were extracted from venous blood, and some were extracted from cell lines or from saliva*.

## The Impact of NF-κB on Different Cell Types

### Macrophages/Microglia

Notably, brain resident microglia, which are developmentally and functionally distinct from blood-borne myeloid cells, are now known to originate from embryonic yolk sac precursors. Because of the phenotypic similarities between microglia, CNS-resident macrophages, and circulating monocyte-derived macrophages, surface markers such as CD11b and F4/80 used by most studies actually stain all these populations (39). Thus, unless otherwise specified, the effects of macrophages/microglia in MS are discussed together.

Prominent macrophage and microglial activation at the site of actively demyelinating plaques is believed to play a central role in MS development (23). Consistent with this, increased nuclear expression of RelA, c-Rel, and NF-κB p50 in macrophages, as well as RelA in microglia, was found to correlate with MS lesion activity (38). Moreover, recent studies have suggested that M1 phenotype macrophages/microglia are crucial in driving EAE progression by secreting large amounts of proinflammatory cytokines and chemokines and reactivating encephalitogenic T cells as antigen-presenting cells (APCs), whereas M2 phenotype macrophages/microglia protect against disease and have a potent ability to release anti-inflammatory molecules and growth factors (40). Researchers found that mice in which *Ikk*β was conditionally knocked out in myeloid cells, which targeted the majority of macrophages and microglia, exhibited ameliorated EAE progression accompanied by reduced levels of macrophage/microglia infiltration, M1 polarization, and CD4^+^ T cell responses (41). Similarly, the silencing of *c-Rel* in macrophages alleviated EAE symptoms through suppressing proinflammatory cytokines and T cell accumulation (42). Depletion of microglial transforming growth factor β-activated kinase 1 (*TAK1*), a molecule that modulates NF-κB, was shown to inhibit the NF-κB canonical pathway and attenuate EAE pathology (43). By contrast, knocking out NF-κB regulatory protein A20 in microglia was associated with massive microglia activation, neuroinflammation, and increased EAE pathology (44). And knocking out IκBα in mouse myeloid cells led to similar findings. These mutant mice displayed constitutively active NF-κB, which led to a prominent increase in macrophages/microglia, T cells, and key proinflammatory mediators (45).

Notably, because the mechanism of NF-κB regulation is not entirely clear, changing certain molecules may result in opposing outcomes. For instance, in contrast to the findings in *IKK*β-deficient macrophages, blocking IKKα was shown to elicit aberrant NF-κB activation that made macrophages hyperactive to various stimuli (46).

### Oligodendrocytes

By immunohistochemistry, strong immunoreactivity for NF-κB p65 was observed in approximately half of the oligodendrocytes in active, but not chronic silent, MS lesions (38). To assess the impact of NF-κB on normal oligodendrocyte maturation, demyelination, and remyelination in an MS background, Raasch et al. depleted IKKβ in CNS cells (IKKβ^CNS-*KO*^) and oligodendrocytes alone (IKKβ^Oligo−KO^) (47). As a result, structurally intact myelin sheaths with similar numbers of oligodendrocyte progenitor cells and mature oligodendrocytes were observed in IKKβ^CNS-*KO*^ and IKKβ^Oligo−KO^ mice compared with their wild-type littermates (47). Further studies revealed comparable degrees of demyelination and remyelination between IKKβ^Oligo−KO^ and control in both EAE and cuprizone models, suggesting that NF-κB in oligodendrocytes is dispensable for myelin loss in patients with MS. However, because IKKβ modulates signaling pathways other than that of NF-κB, such as the extracellular signal–regulated kinase (ERK)-1/2 pathway (48, 49), NF-κB–independent effects on oligodendrocytes cannot be completely ruled out. Similar to mice in which IKKβ had been deleted, mice in which c-Rel, RelB, NF-κB p52, NEMO, or IKKα had been deleted were shown in previous reports to display proper brain myelination under normal conditions (50–52). Notably, NF-κB activity is crucial in orchestrating Schwann cell differentiation and myelination in the peripheral nervous system (PNS), which is in sharp contrast to its role in the CNS; NF-κB activity is crucial in orchestrating Schwann cell differentiation and myelination in the PNS (53).

Furthermore, opinions on the role of NF-κB differ. In a recent study, researchers generated a mouse model that specifically expressed IκBαΔN, a super-suppressor of NF-κB, in oligodendrocytes and documented identical demyelination and remyelination in cuprizone model between IκBαΔN^+^ and IκBαΔN^−^ mice. However, IκBαΔN mice exhibited markedly impaired oligodendrocyte regeneration and remyelination compared to control mice in the presence of interferon γ (IFN-γ) (54). Consistently, IκBαΔN^+^ mice developed much more severe EAE because oligodendrocytes from these mice were more vulnerable to inflammation than those from control mice (54). These data indicate that NF-κB activation may, at least in some circumstances, promote oligodendrocyte survival during inflammation. In contrast, mice in which RelB was absent specifically in oligodendrocytes (RelB^Δ*Oligo*^) showed a decrease in the loss of mature oligodendrocytes, which in turn prevented demyelination upon EAE challenge (55). This protective phenotype was proposed to be the consequence of increased NF-κB p65 activity that protected oligodendrocytes against inflammation. Interestingly, the altered course of EAE in IκBαΔN and RelB^Δ*Oligo*^ mice was more dependent on controlling oligodendrocytes themselves, while the change in inflammation was not significant. Additionally, there was evidence that patients with additional copies of *IKBKG*, the gene encoding NEMO, experience defective CNS myelination due to NF-κB inactivation (52). To date, the effects of NF-κB on oligodendrocytes in normal myelin formation and MS remain ambiguous, and as oligodendrocytes are the main target of MS, further efforts are required to provide clues for future therapeutic approaches.

### Astrocytes

In addition to their role in forming the BBB and supporting neurons, astrocytes are crucial in regulating CNS inflammation (56). Previous reports found nuclear NF-κB p65 in hypertrophic astrocytes in the parenchyma of active MS lesions (38). Recently, Ponath et al. demonstrated that the MS risk variant rs7665090^G^, which is located near *NFKB1*, is associated with upregulated NF-κB and target gene expression (e.g., IFN-γ and TNF-α) in human astrocytes (30). Further characterization revealed stronger immunofluorescent staining for activated NF-κB, chemokines (e.g., CXCL10 and CCL5), and C3d located within the hypertrophic astrocytes and greater perivascular T lymphocyte infiltration in MS lesions with the rs7665090 risk than those with a protective genotype (30). In addition, MS-approved agent fingolimod (FTY720) has exhibited strong anti-inflammatory properties through inhibiting NF-κB activity in astrocytes (57, 58). Therefore, by modulating astroglial NF-κB and thereby relieving the inflamed CNS microenvironment, it is possible to reduce tissue injury and promote later recovery. Consistently, glial fibrillary acidic protein (GFAP)–IκBα-dominant-negative (dn) mice, in which NF-κB was inactivated specifically in astrocytes, manifested alleviated symptoms and steady functional improvement following EAE induction compared to those in wild-type control mice (59). Central nervous system of these mutant animals were characterized by the reduced expression of several proinflammatory cytokines/chemokines and increased levels of CD8^+^ CD122^+^ Tregs, neuroprotective molecules, and unexpectedly, CD45^+^ leukocytes and IL-6 (59). Another study provided similar results based on cuprizone model that the inactivation of astroglial NF-κB dramatically prevented axonal loss through inhibiting proinflammatory cytokines and gliosis in GFAP-IκBα-dn mice (47). Blocking astroglial RelB, however, had a very limited impact on the course of EAE and mainly delayed disease onset with a mild or no effect on CNS inflammation (47). Finally, several adhesion molecules required for BBB integrity, including intercellular cell adhesion molecule 1 and vascular cell adhesion molecule 1, were found to be reduced in GFAP-IκBα-dn EAE mice, further supporting the therapeutic value of interfering with NF-κB in astrocytes.

Although current studies consistently recognize that the activation of astroglial NF-κB exerts detrimental effects on EAE and MS, blocking NF-κB activation may also bring unfavorable outcomes. Many neurotrophic factors released by astrocytes, including nerve growth factor, glial cell line–derived neurotrophic factor, and brain-derived neurotrophic factor, are dependent on the NF-κB pathway (60, 61). Furthermore, IL-6 and leukocytes of GFAP-IκBα-dn EAE mice were significantly upregulated in the CNS (59). Later research conducted by the same group showed, however, a robust reduction in most immune cell populations in the CNS of GFAP-IκBα-dn mice at chronic EAE phase compared with controls (62).

### Neurons

Although neurons generally do not express major histocompatibility complex class I and II molecules and therefore fail to participate in antigen presentation, a growing amount of evidence now suggests that neurons may suppress microglial/microphage activation through the CD220-CD220R interaction, act with T cells to control their survival, and induce the conversion of encephalitogenic T cells to Tregs (63, 64).

Constitutively high basal levels of NF-κB in neurons are essential for regulating cell morphology and plasticity and involved in behavior, learning, and memory (65). Neuronal IKKβ-deficient mice developed a severe, non-resolving form of EAE accompanied by the enhanced accumulation of T_H_1 and NK1.1^+^ cells and proinflammatory cytokines in the CNS (66). In contrast, conditional deletion of the neuronal NF-κB pathway by the transgenic expression of an IκBα super-repressor did not alter the course of EAE (67). One possible explanation for this finding is that IKK ablation, as mentioned above, simultaneously influences other signaling pathways in addition to NF-κB signaling (48). Therefore, other molecules triggered by IKKβ, such as tumor progression locus 2, an important element involved in ERK-1/2 pathways (63), might confer neuroprotection owing to the ability of ERK-1/2 to promote remyelination and decelerate EAE (68, 69). Additionally, the super-repressor was grossly absent in the hindbrain, brainstem, and spinal cord (67), which to some extent restricted the magnitude of neuronal NF-κB deficiency.

### B Cells

Multiple sclerosis has historically been considered a T cell–mediated autoimmune demyelinating disorder. However, an increasing amount of data from neuropathological studies and anti-CD20–directed B cell therapy have made it clear that B lymphocytes also play a critical role in driving inflammation and MS progression (10). B cells have a strong capacity to secrete cytokines and antibodies and reactivate T cells, thereby enhancing immune responses. Further observations indicated that B cells, like T cells, have both pathogenic (effector B cells) and protective (regulatory B cells) effects in the setting of inflammation (70–73).

At present, no *in vivo* studies in an NF-κB–deficient MS model have been performed. It has been reported that a proportion of NF-κB p65 translocated to the nucleus was similar between progressive MS, relapsing MS, and healthy controls (33). By contrast, a recent study revealed that B cells from MS patients exhibited an increased level of activated NF-κB p65 following CD40 stimulation compared with healthy controls (74). The interaction between CD40 and CD40L is a pivotal step in mediating B cell activities (e.g., survival, proliferation, and differentiation) (75). Given the close relationship between aberrant CD40 and autoimmunity (76–78), downregulating the B cell NF-κB pathway, which is a major signaling cascade, by CD40 stimulation may reverse the hyperresponsiveness of B cells induced by CD40 in MS patients. Furthermore, blocking BAFF, a member of the TNF ligand superfamily that specifically regulates B cell functions, was found to promote T cell apoptosis in EAE mice through reducing osteopontin release in an NF-κB–dependent manner (79). The causal MS variant *SP140* (rs28445040-T), which induces decreased SP140 expression, was recently suggested to exerts its function in B cells through upregulating NF-κB activity (80). Finally, the therapeutic mechanism of dimethyl fumarate, which is approved for MS management, has been shown to correlate with a dramatic reduction in proinflammatory B cell subsets *in vitro* partially due to the inhibition of NF-κB activation (81).

In addition, some indirect evidence also points to the benefit of downregulating B cell NF-κB to control excess inflammation. Studies in NF-κB knockout mice have identified distinct functions of different NF-κB proteins. B cells lacking RelB exhibited a proliferation deficit but normal maturation, Ig secretion, and Ig class switching (82). The c-Rel deficiency in B cells was associated with germinal center (GC) collapse and impaired cell growth, whereas RelA deficiency was associated with the weakened development of plasma cells (83). In addition, NF-κB2 deletion mice presented with deficits in antibody secretion, GC reactions, and splenic microarchitecture (84), and NF-κB1 blockade resulted in diminished T cell–dependent antibody responses (85). B cell NF-κB is also essential in preventing autoimmunity, as suggested by de Valle et al., who observed a multiorgan autoimmune disease in NF-κB1 knockout mice that was largely attributable to the dysregulated activity of B cells, which released aberrant levels of IL-6 (86). Overall, because it is difficult to predict whether these immune dysfunctions induced by B cell NF-κB blockade occur in EAE or MS and affect their pathophysiology, further investigations are needed to explore this unidentified issue.

### T Cells

CD3^+^ T lymphocytes are predominant in the demyelinating tissues of patients with MS. However, as CD4^+^ T cells are the major mediators of CNS injury during the course of EAE, in studies of human MS, in which conspicuous CD8^+^ T cells are infiltrated throughout lesions at all stages, whereas CD4^+^ T cells are sparse or even absent, an added challenge for determining the precise role of CD4^+^ T cells is ascertaining whether these effectors are more critical in disease initiation than in established MS (11). And so far, no approved clinical trial that selectively eliminates CD4^+^ T cells has provided definitive evidence of clinical efficacy (87). The significance of T cells and key differences in the inflammatory response between MS patients and EAE animals has recently been reviewed in detail, and caution must therefore be taken when extrapolating animal findings to humans.

There are data, but no direct proof as of yet, associating T cell NF-κB signaling with the risk and maintenance of MS. First, the level of lymphocytic DNA-binding NF-κB p50 was found to be higher in MS patients than in their healthy counterparts, and NF-κB p65, despite its normal level of expression, decreased significantly during therapy (32). In another study, naive CD4^+^ T cells from MS patients were reported to exhibit enhanced activation of NF-κB p65 (34). Moreover, the team identified that the presence of MS risk variant rs228614 proximal to NF-κB1 resulted in increased degradation of IκBα and NF-κB p65 phosphorylation in both TNF-α-stimulated or PMA (phorbol 12-myristate 13-acetate)–stimulated CD4^+^ T cells (34). Similarly, Yan et al. observed that the amount of nuclear NF-κB p65 in CD3^+^ T cells of all MS subgroups was significantly higher compared with healthy controls (33). Second, a large number of MS candidate genes were found to interact in a tractable pathway regulating T_H_1/T_H_17 inflammation, Treg tolerance, and NF-κB induction (88). The imbalance between Treg and T_H_1/T_H_17 cells critically involves in the pathogenesis of EAE and other autoimmune and inflammatory diseases (89, 90). Finally, the data from a network analysis of the CD3^+^ T cell transcriptome implicate aberrant regulation of gene expression by NF-κB as a biomarker of acute MS relapse (35).

In T cells, the mediation of NF-κB subunits by the downstream of T cell receptor mainly requires p50-p50, p50-p65, or p50-c-Rel dimers (91). The importance of NF-κB1 (p50/p105) functioning can be seen in mouse models; NF-κB1–deficient mice develop normally and acquire a structurally normal immune system but are resistant to EAE. Further investigation suggested that this protection was due to the hindered activation and differentiation of MOG-specific T_H_1 and T_H_2 cells in these mutant EAE mice (92). Notably, as NF-κB p105 also belongs to the IκB protein family, previous studies have noted a remarkable increase in the activation of CD4^+^ T cells and a higher frequency of memory/effector T cells in mice specifically deficient for p105 compared to wild-type mice (93). Nuclear factor κB1, however, may also have beneficial effects because NF-κB1–deficient EAE mice showed more infiltrated inflammatory cells in the CNS than control mice. Consistently, p50-deficient mice exhibit augmented microglial proinflammatory responses after peripheral injection with lipopolysaccharide (94).

Although *c-Rel* knockout mice do not suffer from development defects, studies have clarified that T cells from these mice are impaired in their ability to activate and generate cytokines such as IL-2, IL-3, and granulocyte–macrophage colony-stimulating factor and differentiate into effector populations (95–97). Similar to mice lacking NF-κB1, mice lacking c-Rel were shown to be protected from EAE (98). In contrast to NF-κB1–deficient mice, in which T_H_2 cell differentiation was preferentially compromised (99), splenocytes derived from c-Rel–deficient mice produced undetectable IFN-γ and increased levels of IL-4 (98), denoting their non-overlapping capacities for different NF-κB molecules. Later *in vitro* studies led to the conclusion that c-Rel–deficient CD4^+^ T cells are intrinsically unable to generate IFN-γ under both T_H_0- and T_H_1-polarizing conditions (98). Moreover, *c-Rel*–deficient APCs displayed a substantially reduced level of IL-12 p40, an essential cytokine for T_H_1 cell differentiation, which further aggravated T_H_1 cell deficiency (98). In addition to promoting T_H_1 cells, c-Rel is involved in the development of T_H_17 cells through directly controlling expression of the *Rorc* gene, which encodes the T_H_17 cell–specific transcription factor retinoic acid–related orphan receptor γt, and indirectly facilitating APCs to generate IL-23, a critical molecule known to enhance IL-17 expression by CD4^+^ T cells (97, 100, 101). Consistent with these findings, the inability to provoke optimal T_H_1 and T_H_17 cell immune responses occurred in parallel with an ameliorated phenotype in *c-Rel* knockout mice after EAE induction (98, 100). Moreover, c-Rel may additionally influence cytotoxic T lymphocytes because cell survival was dramatically impaired in c-Rel-deficient CD8^+^ T cells that could be reversed with IL-2 addition (102); however, the capacity of c-Rel-deficient CD8^+^ T cells to clear viral infection was not affected (103). Although c-Rel deficiency confers resistance to several T cell–dependent autoimmune disorders such as EAE and collagen-induced arthritis (100, 104), novel data have demonstrated the anti-inflammatory effect of c-Rel in promoting the Treg cell lineage, as revealed in *c-Rel*–deficient mice, in which thymic and peripheral CD4^+^ Foxp3^+^ T cells were vastly reduced in number compared to wild-type counterparts (105, 106). Defects in Treg cells are now thought to be partially due to the direct regulation of *Foxp3* enhanceosomes by c-Rel (107). Furthermore, as the addition of exogenous IL-2 was sufficient to rescue Foxp3 deficiency, decreased IL-2 generation in *c-Rel* deletion mice may amplify the lack of Treg cell expansion (106). Notably, despite their decreased frequency, *c-Rel*-deficient Tregs were indicated by *in vitro* and *in vivo* findings as capable of suppressing effector T cells at normal ranges (105).

There are no data exploring the changes in EAE under *NF-*κ*B p65*-deficient conditions because of embryonic lethality and liver degeneration (108). Lymphocytes derived from SCID (severe combined immunodeficient) mice transplanted with *p65*-deficient fetal liver cells displayed normal development and IL-2 expression but were defective in their proliferative response to various mitogens (108). Moreover, p65 deficiency in T cells largely blocked T_H_17 cell differentiation in a manner similar to that of c-Rel deficiency, which was caused by reduced *Rorg* activity in the T_H_17 cell lineage (101). In contrast, studies utilizing T cell–specific *p65* mutant mice have indicated that p65 is dispensable in T_H_17 cell differentiation but required for another important source of IL-17, γδ T cells (109). On the other hand, p65 might prevent EAE with its potent capacity to modulate Treg cell homeostasis. In recent years, mounting evidence has identified p65 as an essential component in mature Treg cell identity formation, tolerogenic function, and egress from the thymus (110–112). Finally, in addition to c-Rel, T_H_1 differentiation is also regulated by RelB. The absence of RelB in T cells led to a dramatic decrease in T_H_1 differentiation and IFN-γ production, but the conventional T_H_17 polarization was normal.

## Therapeutic Potential For MS

The therapeutic efficacies of many approved treatments for MS are now thought to be attributed, at least in part, to blockade of NF-κB pathway of the peripheral nervous system and CNS immune response. Dimethyl fumarate, for instance, was shown to effectively inhibit the generation of IL-6, TNF, nitric oxide (NO), and NF-κB activation in stimulated microglia, and its active metabolite, monomethyl fumarate, was found to suppress myeloid dendritic cell (DC) maturation partially *via* NF-κB signaling, hence reducing proinflammatory activities in cocultured T cells (113–115). The effects of FTY720 and phosphorylated FTY720 were observed to decrease NF-κB activity in cultured astrocytes (57, 58). And the most widely used glucocorticoids were found to downregulate NF-κB through both directly inhibiting p65-dependent gene activation and indirectly enhancing IκBα synthesis (116, 117). Responsiveness to laquinimod, a novel immunomodulatory compound for relapsing-remitting MS, was linked to its ability to impair DC maturation and function through NF-κB interference (118). Moreover, laquinimod was reported to ameliorate CNS inflammation and myelin loss in a cuprizone model by attenuating astrocytic NF-κB activation (119).

Although no NF-κB inhibitors have been approved to the clinical treatment for MS, the beneficial effects of NF-κB interference by a considerable number of natural components (e.g., piperlongumine and denanthin) have been suggested in basic animal studies (120, 121). Moreover, the selective NF-κB inhibitor pyrrolidine dithiocarbamate markedly alleviated the incidence and severity of EAE in rats (122). The IKK1/2 inhibitor reduced plasma IL-17 and IFN-γ levels and reduced EAE symptoms (123). Administration of PS-1145, a compound that inhibits IKK2- and NEMO-dependent canonical NF-κB signaling but maintains the alternative NF-κB signaling pathway, effectively improved EAE, which was characterized by decreased lymphocytic proliferation and cytokine (IL-2 and IL-17) production (124). Similarly, peptides corresponding to the NEMO-binding domain displayed a potent propensity to suppress encephalitogenic T cell generation and activation and T_H_1 cell responses, thus significantly protecting against EAE (125). Finally, a novel peptide from glucocorticoid-induced leucine zipper (GILZ), a molecule that binds and inhibits NF-κB p65, was shown to increase the level of IL-10 and reduce IFN-γ, IL-12, and IL-17 levels in GILZ-treated EAE mice (126).

As discussed above, excessive and persistent immune reactions primarily contribute to MS tissue injury. This context gives rise to uncontrolled NF-κB activity, which further drives ongoing inflammation in a self-amplifying cycle. Several lines of evidence have highlighted the beneficial effects of NF-κB pathway inhibition based on clinical and basic data. However, because basal NF-κB is crucial to normal cellular physiology and pathogen clearance, the non-selective blockade of NF-κB may therefore lead to many unwanted side effects, as we recently reported that the blockade of TNFR1 or TNFRII had a completely difference consequence on T_H_17 and Treg cells (127). Furthermore, as NF-κB exerts diverse effects depending on the isoform member, type of activated cells, and strength of the triggering event, it would be difficult to predict the therapeutic outcome. Another hurdle lies in the differences between the EAE model and human MS, as well as the heterogeneous pathogenesis in patients with relapsing and progressive MS (11). As such, great effort has been made to increase the safety of NF-κB interference, including selectively diminishing NF-κB activity by targeting the IKK complex, IκB proteins, and the ubiquitin–proteasome system (124, 125). Organ-specific NF-κB interference has raised much attention owing to its potential effect to avoid systemic side events. Local administration of NF-κB decoy oligodeoxynucleotides (ODNs) encapsulated in a viral vesicle was shown to treat various models of inflammatory colitis without impairing extraintestinal NF-κB activation (128). Further study on the delivery of a naked NF-κB decoy ODN to inflamed tissue also indicated success in improving murine bowel disease and restoring colon homeostasis (129).

## Conclusion

Multiple sclerosis is an autoimmune inflammatory disease driven by the complex interaction between environment and predisposing genes. Compelling data support the critical role of aberrant NF-κB activation, which triggers proinflammatory activities via multiple aspects, in the pathogenesis of MS and EAE. As NF-κB has both beneficial and detrimental effects, promising agents have been explored to retain essential NF-κB activity. In this regard, a better understanding of the molecular events that determine the point at which NF-κB responses switch from being protective to mediating damaging effects is needed for the therapeutic modulation of neuroinflammation and neurodegeneration.

## Author Contributions

YZ completed the first draft. WQ and SZ reviewed and improved this manuscript. CC, WL, and XM polished the article.

### Conflict of Interest

The authors declare that the research was conducted in the absence of any commercial or financial relationships that could be construed as a potential conflict of interest.
